# Genomic and temporal analyses of *Mycobacterium bovis* in southern Brazil

**DOI:** 10.1099/mgen.0.000569

**Published:** 2021-05-20

**Authors:** Rudielle de Arruda Rodrigues, Flábio Ribeiro Araújo, Alberto Martín Rivera Dávila, Rodrigo Nestor Etges, Julian Parkhill, Andries J. van Tonder

**Affiliations:** ^1^​ Postgraduate Program in Veterinary Science, Faculty of Veterinary Medicine and Animal Science, Federal University of Mato Grosso do Sul, Campo Grande, Brazil; ^2^​ Embrapa Beef Cattle, Campo Grande, Brazil; ^3^​ Computational and Systems Biology Laboratory, Graduate Program in Biodiversity and Health, Oswaldo Cruz Institute, Fiocruz, Rio de Janeiro, Brazil; ^4^​ Secretariat of Agriculture, Livestock and Irrigation, Porto Alegre, Brazil; ^5^​ Department of Veterinary Medicine, University of Cambridge, Cambridge, UK

**Keywords:** bovine tuberculosis, livestock, whole genome sequencing, transmission, Brazil

## Abstract

*
Mycobacterium bovis* is a causal agent of bovine tuberculosis (bTB), one of the most important diseases currently facing the cattle industry worldwide. Tracing the source of *M. bovis* infections of livestock is an important tool for understanding the epidemiology of bTB and defining control/eradication strategies. In this study, whole genome sequencing (WGS) of 74 *M *. *bovis* isolates sourced from naturally infected cattle in the State of Rio Grande do Sul (RS), southern Brazil, was used to evaluate the population structure of *M. bovis* in the region, identify potential transmission events and date the introduction of clonal complex (CC) European 2 (Eu2). *In silico* spoligotyping identified 11 distinct patterns including four new profiles and two CCs, European 1 (Eu1) and Eu2. The analyses revealed a high level of genetic diversity in the majority of herds and identified putative transmission clusters that suggested that within- and between-herd transmission is occurring in RS. In addition, a comparison with other published *M. bovis* isolates from Argentina, Brazil, Paraguay and Uruguay demonstrated some evidence for a possible cross-border transmission of CC Eu1 into RS from Uruguay or Argentina. An estimated date for the introduction of CC Eu2 into RS in the middle of the 19th century correlated with the historical introduction of cattle into RS to improve existing local breeds. These findings contribute to the understanding of the population structure of *M. bovis* in southern Brazil and highlight the potential of WGS in surveillance and helping to identify bTB transmission.

## Data Summary

1. Supplementary files 1 and 2 (available in the online version of this article).
2. Raw sequencing data for all sequenced isolates have been deposited in the European Nucleotide Archive (ENA) under project PRJEB39667. Accession numbers are detailed in File S1.
3. The R code used to analyse the data in this paper is available on Github: https://github.com/avantonder/Brazil2020.

**Impact Statement**

*
Mycobacterium bovis* is a causal agent of bovine tuberculosis (bTB), one of the most important diseases currently facing the cattle industry worldwide. Tracing the source of *M. bovis* infections of livestock is an important tool for understanding the epidemiology of bTB and defining control/eradication strategies. This is, to date, the largest whole genome sequencing-based study of *M. bovis* in Brazil and South America and will be of interest to many researchers in the bacterial genomics field. Our study highlighted the diversity of *M. bovis* in southern Brazil, identified potential within- and between-herd transmission often over large distances, and estimated a potential date of introduction of clonal complex Eu2 into the region that correlates with the historical introduction of cattle into the State of Rio Grande do Sul to improve existing local breeds. Further, the work showed that even with limited sampling, genome sequencing has the potential to be a useful tool for helping to control bTB transmission in Brazil.

## Introduction

*Mycobacterium bovis* is a causative agent of bovine tuberculosis (bTB), infecting mainly bovines globally, in addition to a broad host range that includes humans and wild animals. Moreover, the disease in humans constitutes a public health problem and currently is one of the most important diseases faced by the global cattle industry, causing significant economic losses to beef and dairy production [1–3].

In many countries, national control and eradication bTB programmes have been successfully implemented in livestock, based on test and slaughter of infected cattle as a strategy to manage the disease [1, 2]. In Brazil, which is currently the world’s largest exporter of beef [4], the disease is compulsorily reported and regulated by the National Program for the Control and Eradication of Animal Brucellosis and Tuberculosis (PNCEBT), which aims to reduce the prevalence and incidence of bTB in cattle and buffaloes, by intradermal testing and the slaughter of reactive animals, and eventually aims to eradicate bTB in Brazil [5]. In the State of Rio Grande do Sul (RS), since December 2014, the Normative Instruction of the Secretariat of Agriculture, Livestock and Agribusiness no. 002/2014 (IN-SEAPA 002/2014) has worked with the objective of complementing the actions of the PNCEBT, avoiding the dissemination of diseases, as well as promoting the process of detection of outbreaks through the diagnosis of disease by qualified veterinarians from the programme [6].

Official epidemiological surveys of bTB performed in 75 % of Brazilian herds show that the prevalence of this disease is heterogeneous among and within states with prevalence of infected herds ranging between 1.0 and 13.9 %, prevalence being higher in dairy farms, especially high-production farms [7]. The main risk factors identified were milk production, particularly in farms with some degree of sophistication in the mode of production, and having a larger number of cattle [8–20].

In southern Brazil, RS currently has the seventh largest cattle population, with 12 918 325 animals (estimated in 2019), which represents 6.05 % of the Brazilian national herd [21]. The state stands out in national milk production, being the second largest producer with about 13.2 % of national production [22]. Study of the epidemiological situation of bTB in RS reported the prevalence of outbreaks to be 2.8 % [95 % Confidence Interval (CI): 1.8–4.0 %] at the herd level and 0.7 % [95 % CI: 0.4–1.0 %] at the animal level. It was found that there was a concentration of outbreaks in the northern part of the state, characterized by the predominance of milk and mixed beef–dairy farms. The risk factors associated with the outbreak condition were dairy farming (odds ratio, OR=2.90 [95 % CI: 1.40–6.13]) and herds with 16 or more cattle at least 24 months old (OR=2.61 [95 % CI: 1.20–5.49]) [15]. The PNCEBT-RS statistics recently reported that the prevalence of outbreaks was 1.9 % and that of cases was 1.0 % [23].

Genetic differentiation of *M. bovis* strains can provide better understanding about the evolution, epidemiology and ecology of bTB [24–27]. The molecular tools currently used worldwide for genotyping *M. bovis* isolates are spoligotyping and variable number tandem repeat (VNTR) analysis [28–31]. However, although these genotyping methods have proven useful in identifying local clusters, there are limitations regarding their ability to discriminate events within clusters involved in persistence and in assessing the spread of bTB [32, 33].

On the other hand, whole genome sequencing (WGS) has been successfully used to study multi-scale bTB epidemiology [26, 34, 35], offering much higher resolution than other established typing methods, which greatly improves the definition of the regional location of *M. bovis* strains [33, 36]. Among *M. bovis* WGS studies, many have focused on investigating bTB transmission and source within livestock populations [32, 37–40].

The first complete Brazilian *M. bovis* genome, SP38, was published in 2017 and belonged to clonal complex (CC) European 2 (Eu2) [41]. Using WGS-based multi-locus sequencing typing, a recent study showed evidence of *M. bovis* transmission events between farms and multiple introductions into a specific farm in the state of Santa Catarina, southern Brazil [42]. Another recent WGS study in northern Brazil has reported an endemic and unique *M. bovis* clade responsible for ongoing livestock transmission events in both cattle and buffalo [35].

In this study, 74 *M *. *bovis* isolates, sampled from naturally infected cattle taken from areas of occurrence of bTB in RS, southern Brazil, were sequenced. The aims of the study were to characterize the population structure and diversity of *M. bovis* in RS, attempt to identify potential transmission events over short and long distances, and gain insights into the use of a WGS-based surveillance programme to improve control of bTB in the cattle population in RS. Additionally, a global collection of 316 CC Eu2 isolates, including 64 from this study, was used to date the most recent common ancestor (MRCA) of this lineage and estimate the date of introduction of Eu2 into Brazil.

## Methods

### Sample collection

All of the *M. bovis* isolates were obtained from naturally infected dairy and beef cattle, detected using comparative intradermal tuberculin testing (CITT) according to the rules of the PNCEBT [5], from bTB-positive farms (outbreaks) in the period between 2015 and 2019 by the Secretariat of Agriculture, Livestock and Irrigation (SEAPI). Farms included in this study were chosen if they had a history of bTB, were geographically proximate and/or had a history of animal movements between them. For each infected animal, samples were collected from each lesion suggestive of tuberculosis (LST) at the same time point; for animals with multiple lesions, samples corresponding to each tissue type were cultured. Samples were collected from tissue (lung, liver, carcass) and lymph nodes (submandibular, pulmonary, parotid, mediastinal, mesenteric, retropharyngeal and tracheobronchial; File S1). Positive animals based on CITT were subjected to sanitary slaughter, according to Brazilian legislation. A total of 196 samples were collected from 78 animals from 13 farms.

### Isolation and identification of *M. bovis*

All tissue samples with or without LST were macerated with 1.5 ml of sterile distilled water in a Magna Lyser apparatus (Roche Life Science), decontaminated by the Petroff method [43], subjected to cultivation in Stonebrink culture medium and incubated at 37 °C for up to 90 days. Confirmation of the isolates was performed by conventional PCR with primers that flank the differentiation region 4 (RD4), absent in this species but present in all other members of the *M. tuberculosis* Complex (MTBC) [44]. DNA extractions for genomic sequencing from the cultured *M. bovis* isolates were performed according to the protocol of van Embden *et al*. [45], with modifications (File S2). A total of 76 isolates were successfully cultured and sent for sequencing.

### Sequencing and genomic analysis

The isolates were sequenced using the Illumina NextSeq 500 and HiSeq 2500 platforms. Raw sequence reads were trimmed using TRIMMOMATIC-v0.32 [46] and the species composition of the reads was assessed using Kraken [47] and Bracken [48]. Samples with fewer than 70 % *Mycobacterium* reads were excluded (*n*=2). The trimmed sequence reads were mapped to the *M. bovis* AF2122/97 reference genome (NC0002945) using the Burrows-Wheeler Aligner (BWA; minimum and maximum insert sizes of 50 and 1000 bases) [49]. SNPs were processed with SAMtools mpileup and BCFtools (minimum base call quality of 50 and minimum root squared mapping quality of 30) [50, 51]. Genomic regions consisting of GC-rich sequences such as PPE proteins and PE-PGRS repeats were masked in the resulting alignment using previously published coordinates [52]. Variant sites in the alignment were extracted using snp-sites [53] and a maximum-likelihood phylogenetic tree was reconstructed using IQ-tree [54] accounting for constant sites in the alignment and run for 1000 bootstraps using the extended model selection function. The resulting tree was rooted using the AF2122/97 reference and annotated in iTOL [55]. Genomic clusters were assigned using fastBAPS [56] and pairwise SNP distances were calculated for all pairs of isolates using pairsnp (https://github.com/gtonkinhill/pairsnp).

For the detection of *in silico* spoligotypes of *M. bovis *, SpoTyping-v2.1 [57] was run on the raw sequence reads. The binary code representation of the spoligotypes was compared against the International *Mycobacterium bovis* Spoligotype Database (www.mbovis.org) to assign the spoligotype (SBXXXX) to each isolate [58]. RD-analyzer [59] was used to assign the CCs to the isolates (File S1).

### Statistical analysis

The R [60] library ggmap [61] was used to construct a map showing the geographical locations, based on the latitude and longitude of each farm sampled, of each sequenced isolate. The same geographical coordinates were plotted using the R library ggplot [62] and a convex hull, calculated using the getConvexHull function from the R library contoureR [63] and representing the smallest polygon incorporating a particular set of points, was drawn around isolates from each fastBAPS cluster. The distHaversine function from the R library geosphere [64] was used to calculate the pairwise geographical distance in kilometres between each isolate. Mantel tests (1000 permutations to assess significance) were implemented using the R library ade4 [65–68] to test for an association between genetic and spatial distance. Putative transmission clusters were defined using two different pairwise SNP thresholds (15 SNPs to capture older transmission events and five SNPs to identify more recent transmission) using the R library iGRAPH [69]. The networks were then plotted with ggraph [70]. All isolates regardless of which animal they were collected from were treated as independent samples.

### Contextual and dating analysis

To provide regional context for the 74 isolates sequenced in this study, sequence data for 35 published isolates from Brazil and Uruguay [35, 39, 41], and assemblies for 18 isolates from Argentina, Brazil and Paraguay [24, 71] (File S1) were downloaded from the European Nucleotide Archive (ENA) and the NCBI respectively. The assemblies were converted to fastq files using fastaq (https://github.com/sanger-pathogens/Fastaq) and the resulting data were processed as above.

In order to estimate a date for the introduction of CC Eu2 into RS, a global collection of 252 published Eu2 isolates was assembled [25, 40, 41, 72–76] (File S1). Quality control and mapping were performed as above and the isolates were combined with the 64 Eu2 isolates sequenced in this study. The presence of temporal signal in the Eu2 dataset was investigated by plotting the root to tip distance for each isolate, calculated using the R library phytools [77], against its sampling date (Fig. S1). The slope, *x*-intercept (MRCA), correlation coefficient and *R* ^2^ value were calculated in R. The Eu2 mapped alignment was used as input for BEAST v1.8.4 [78] and the tip sampling dates were used for calibration. Three runs of 10^8^ Markov chain Monte Carlo (MCMC) iterations were performed using an HKY substitution model, strict or constant molecular clock, and constant or exponential population size and growth (12 separate runs). The performance of each model was assessed through the comparison of posterior marginal likelihood estimates (MLEs) and the model with the highest Bayes factor [79] (strict clock/constant population size) was selected (Table S1). The three MCMC runs were combined using LogCombiner v1.8.4 (10 % burnin) and convergence was assessed [posterior effective sample size (ESS) >200 for each parameter] using Tracer v1.6. A maximum clade creditability tree summarizing the posterior sample of trees in the combined MCMC runs was produced using TreeAnnotator v1.8.4 and the resulting tree was annotated using ggtree [80]. In order to confirm the temporal signal in the dataset, the R library TIPDATINGBEAST [81] was used to generate 20 new datasets with randomly assigned dates; BEAST was then run on these new datasets using the same strict constant priors. As the estimated substitution rates in the observed data did not overlap with the estimated substitution rates in the randomized data, the temporal signal observed in the observed data was not obtained by chance (Fig. S2).

## Results

### Dataset characterization

**Fig. 1.**
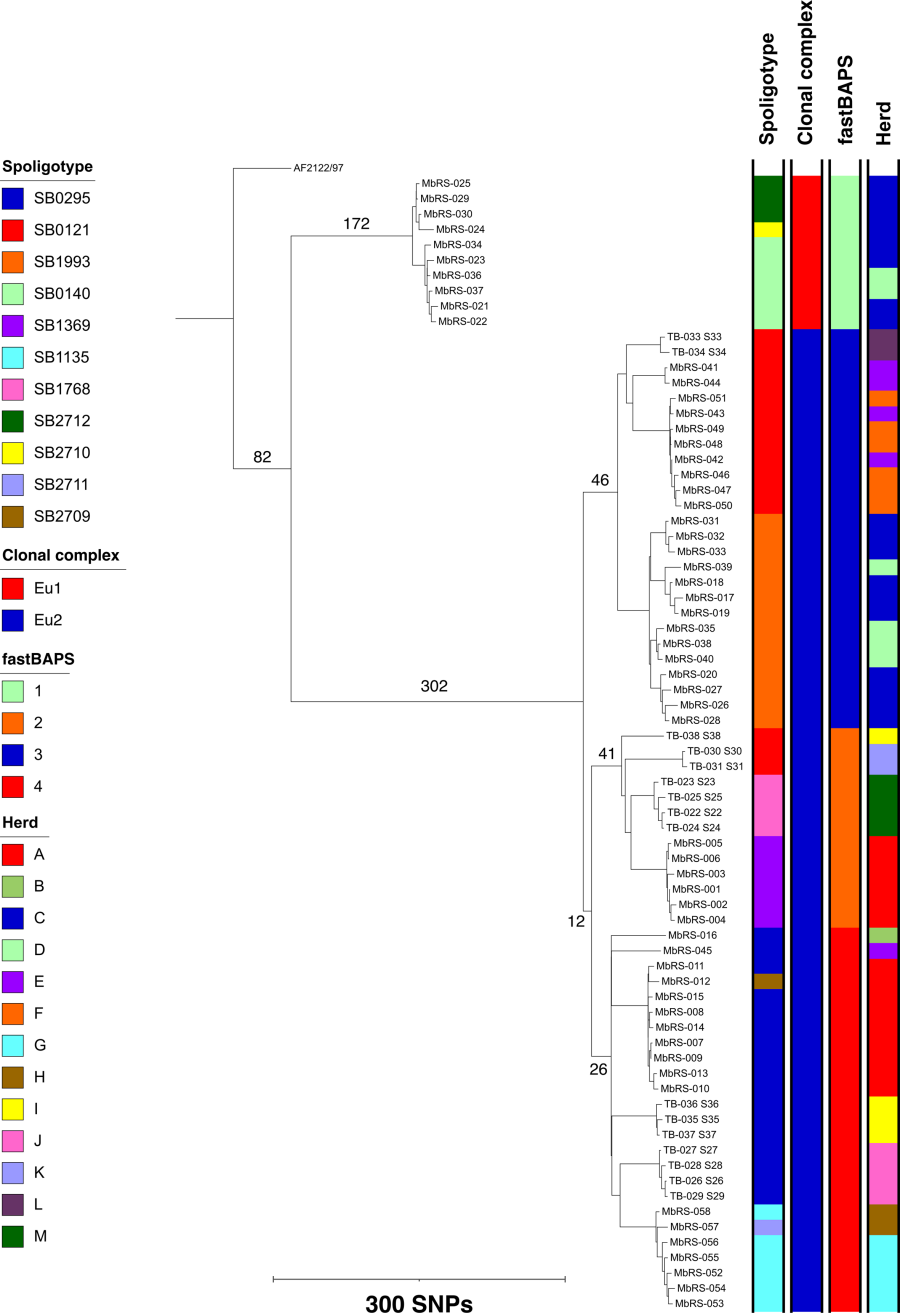
Maximum-likelihood phylogenetic tree of 74 *M. bovis* isolates from Rio Grande do Sul, Brazil. The tree was rooted using the AF2122/97 reference. Branch lengths for the major branches are given in number of SNPs.

In total, 74 *M. bovis* isolates were sequenced having been obtained from the bacteriological culture of tissues of 56 cattle with or without LST. All colonies indicative of *M. bovis* were confirmed by conventional PCR for the RD4 flanking region. Isolates were obtained from 13 distinct herds (A–M) across three mesoregions in the state of RS, Brazil (Fig. 1). Genotypic characterization by *in silico* spoligotyping revealed seven previously identified profiles: SB0295 (*n*=17), SB0121 (*n*=15), SB1993 (*n*=14), SB1369 (*n*=6), SB1135 (*n*=6), SB0140 (*n*=6) and SB1768 (*n*=4). Four new spoligotypes were defined: SB2709 (*n*=1), SB2710 (*n*=1), SB2711 (*n*=1) and SB2712 (*n*=3). Isolates with two different CCs, European 1 (Eu1) and Eu2, were present in the dataset with the majority (64/74, 86.5 %) classified as Eu2 (File S1). The phylogenetic tree of the 74 Brazilian isolates, rooted with AF2122/97 as a reference (CC Eu1), showed a distinct phylogenetic structure with the two included CCs clearly segregating (Fig. 1). Sequence-based clustering using fastBAPS showed that there were four distinct clusters within the phylogeny: 1 (*n*=10), 2 (*n*=13), 3 (*n*=26) and 4 (*n*=25), with cluster 1 composed of isolates from Eu1 and clusters 2–4 comprising isolates from Eu2 (Fig. 1).

### Spatial distribution of fastBAPS clusters

**Fig. 2.**
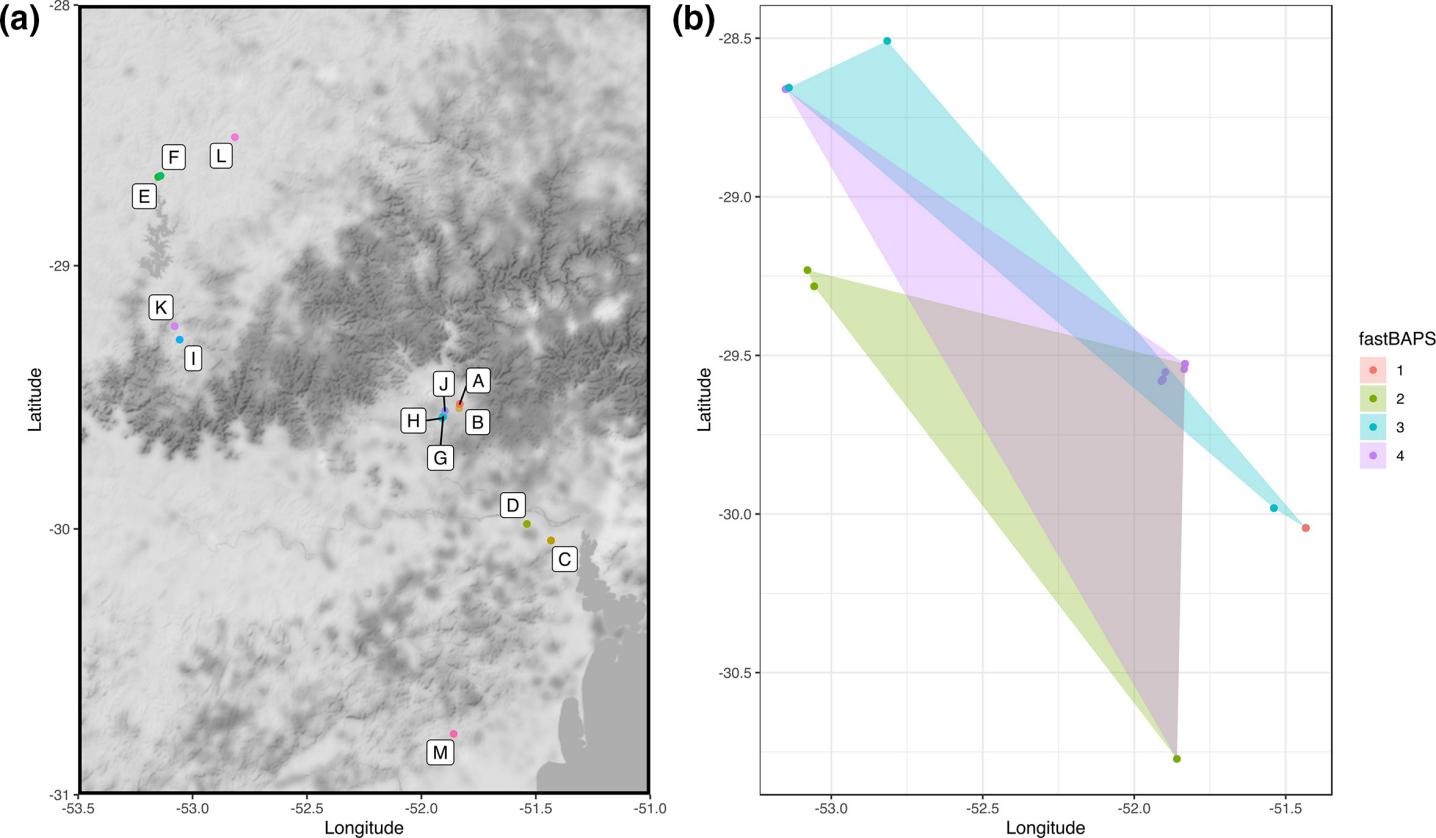
(a) Location of the 13 farms in Rio Grande do Sul sampled in this study. (**b**) Spatial analysis of the distribution of isolates coloured by fastBAPS cluster. Each polygon represents the minimum convex polygon of the sampled locations of the isolates from each fastBAPS cluster.

The locations of the 13 farms sampled in this study are shown in Fig. 2(a). The geographical distribution of the 74 sampled isolates was overlaid with polygons representing the spatial distribution of each fastBAPS cluster (Fig. 2b). These spatial distributions overlapped and could not be separated on the basis of sampling location, showing evidence of multiple *M. bovis* lineages circulating in the region.

### Genetic diversity

**Fig. 3.**
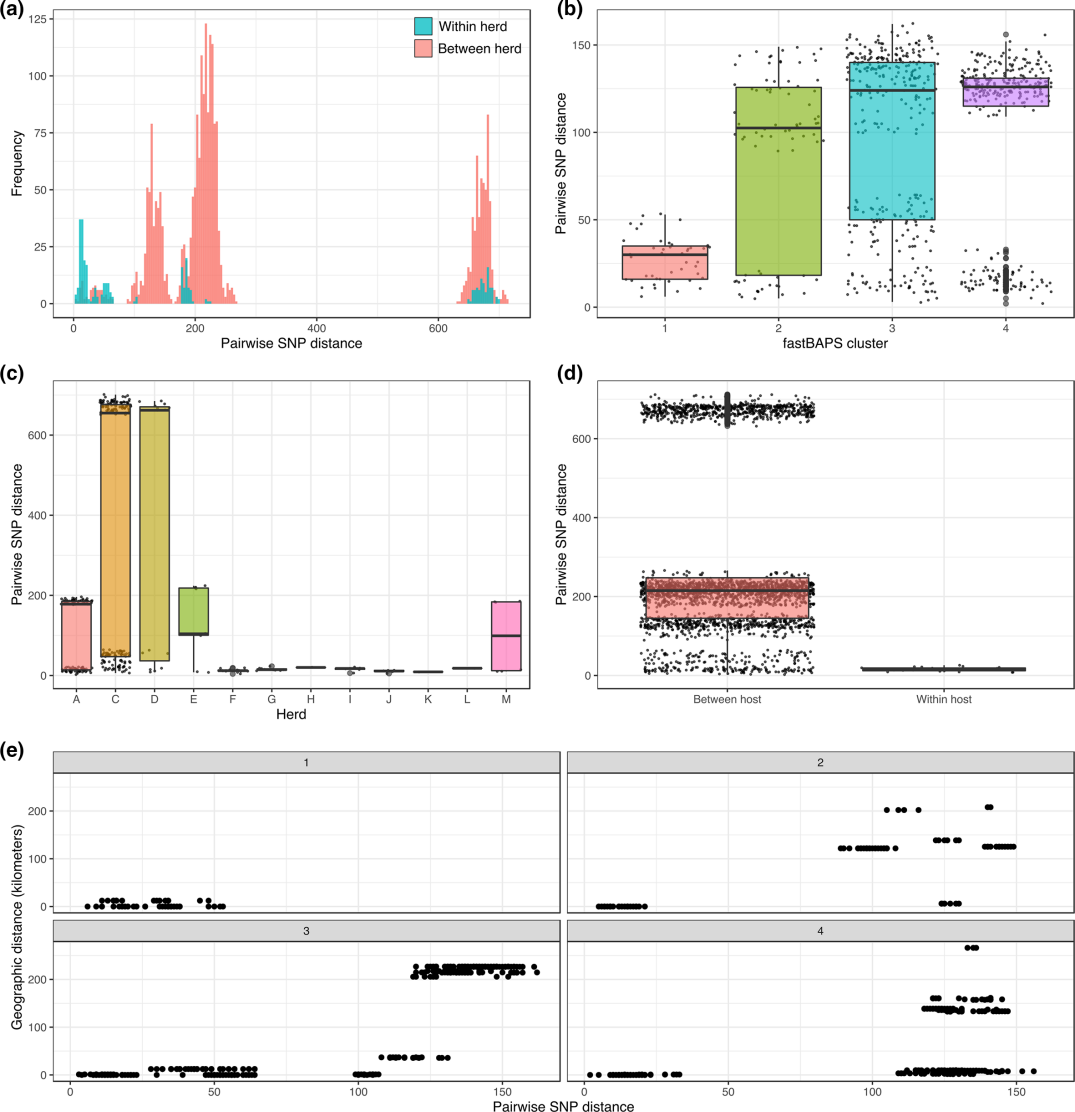
(a) Histogram of pairwise SNP distances separated by between- and within-herd isolate pairwise distances. (**b**) Boxplot of all pairwise SNP distances separated by fastBAPS cluster. (**c**) Boxplot of all pairwise SNP distances separated by herd. (**d**) Boxplot of all pairwise SNP distances separated by within and between animal. (**e**) Scatterplots of pairwise SNP distance against geographical distance for all pairs of isolates separated by fastBAPS cluster.

A multimodal distribution was observed for pairwise SNP distances within the dataset, with the distinct transmission modes representing the population structure of the isolates (Fig. 3a). There were no differences in the distribution of pairwise SNP distances between isolates from the same herd or isolates from different herds, confirming the presence of multiple lineages circulating within and between different herds in the region. Similar levels of pairwise SNP diversity were observed for fastBAPS clusters 2, 3 and 4, with the maximum number of pairwise SNPs between isolates in these clusters ranging between 149 and 162 SNPs (Fig. 3b). By comparison, there was much less diversity within fastBAPS cluster 1, with all isolates being within 53 SNPs of each other. Fig. 3(c) shows the distribution of pairwise SNP distances binned by herd; a high level of diversity was observed for farms C and D, reflecting the presence of both CCs in animals from these herds, whilst a medium level of diversity was seen in herds A, E and M. The remaining herds, F–L, showed much lower levels of diversity, which is probably a reflection of the small number of isolates collected from these herds. The median number of SNPs between isolates from the same animal was 15 and ranged from eight to 26, whilst the number of SNPs between isolates from different animals varied between two and 712 with a median of 215 (Fig. 3d). Fig. 3(e) shows pairwise SNP distances plotted against geographical distances for all pairs of isolates separated by fastBAPS clusters. The result of the Mantel test for each fastBAPS cluster showed that there was no association between genetic and spatial distance for fastBAPS clusters 1 and 4, with a weak association detected for cluster 2 and a stronger association detected for cluster 3 (Table 1).

**Table 1.** Mantel test association scores for each fastBAPS cluster Observed correlations close to 1 represent a strong association between spatial and genetic distance

fastBAPS cluster	Observed correlation	Simulated *p*-value
1	−0.01857867	0.615
2	0.5263921	0.001
3	0.7324717	0.001
4	−0.02190707	0.803

### Genomic epidemiology and transmission

**Fig. 4.**
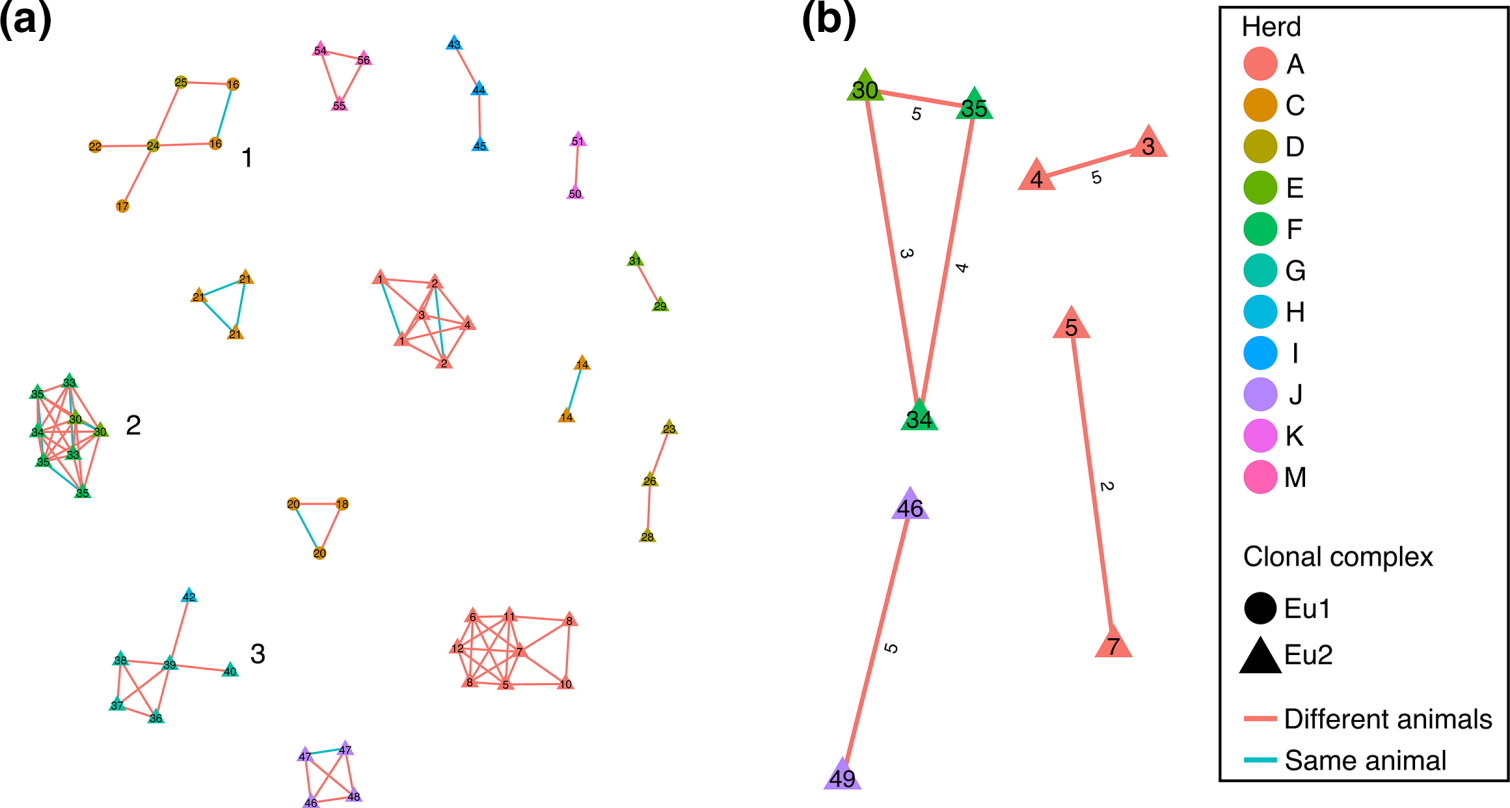
(a) Putative transmission clusters generated using a pairwise SNP threshold of 15 SNPs and clusters with between-herd transmission events (clusters 1, 2 and 3) are labelled. (**b**) Putative transmission clusters generated using a pairwise SNP threshold of five SNPs. The pairwise SNP distances between each pair of isolates is shown. Nodes are coloured by herd and labelled with the animal ID. Clonal complexes are shown by different shapes and links between nodes are coloured according to whether the nodes were from the same animal or not.

A total of 14 putative transmission clusters were defined using the conservative pairwise SNP threshold of 15 and varied in size between two and eight isolates with 15 isolates not part of a cluster; two of the clusters comprised solely of isolates from the same animal (Fig. 4a). The minimum within-herd pairwise SNP distance for the clusters varied between two and 14 SNPs. Only three of the putative clusters contained between-herd links, implying longer distance transmission: cluster 1 (herds C and D; 12.3 km apart), cluster 2 (herds F and G; 157.8 km apart) and cluster 3 (herds G and H; <1 km apart), with the remaining clusters all containing isolates from the same herd. A closer inspection showed that the minimum pairwise SNP distance for isolates from different herds in these clusters was 11, three and 14 SNPs for clusters 1, 2 and 3, respectively.

When the more stringent pairwise SNP threshold of five SNPs was applied, the number of putative transmission clusters dropped to four; 65 isolates were not assigned to a cluster (Fig. 4b). Only one of the three clusters contained links between different herds and was made up of isolates found in cluster 2 identified using the 15-SNP threshold. The remaining three clusters were each made up of two isolates from different animals in the same herds (herds A and J).

### Comparison with other published *M. bovis* isolates

**Fig. 5.**
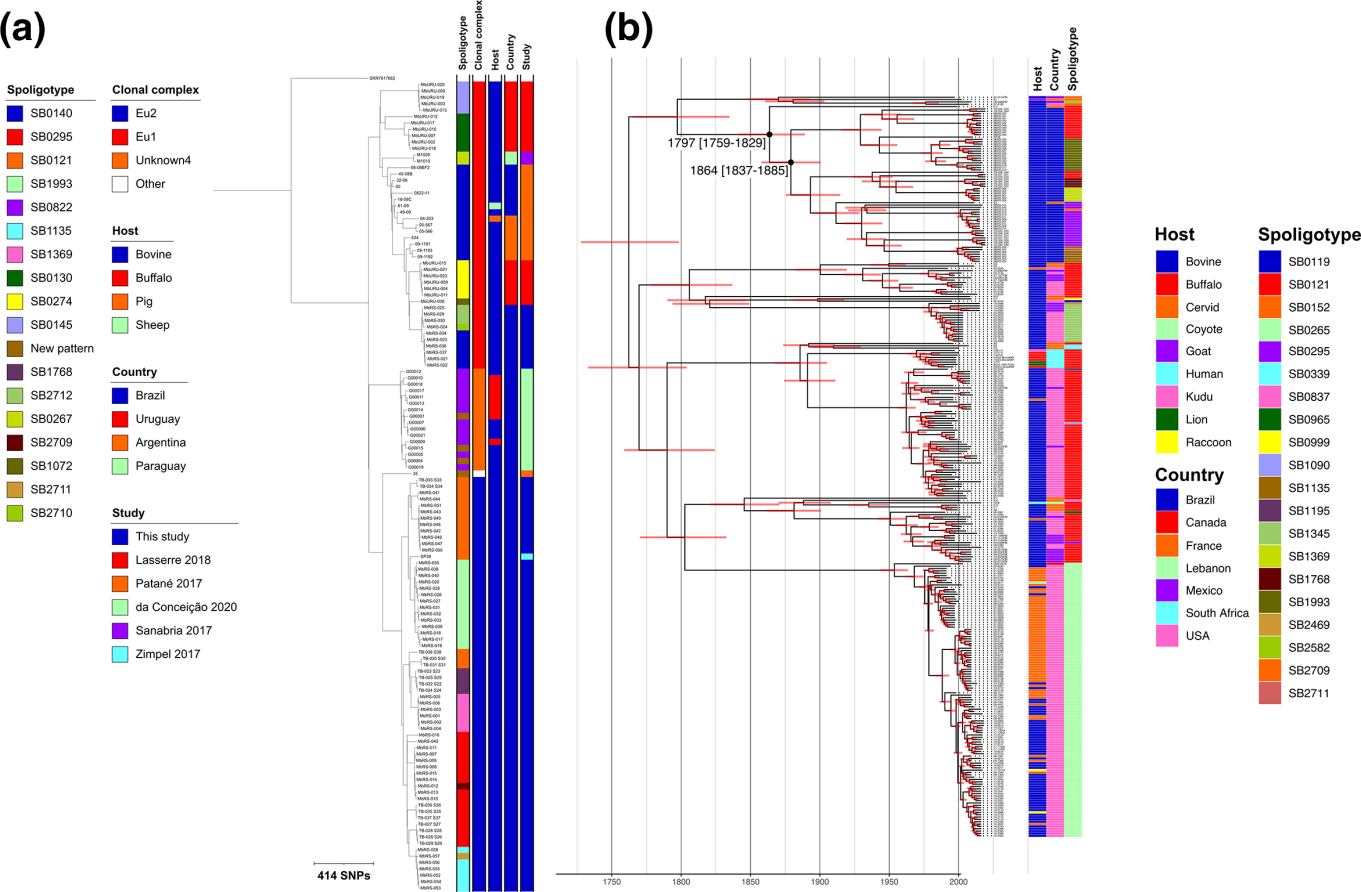
(a) Maximum-likelihood phylogenetic tree of 127 South American *M. bovis* isolates. The tree was rooted using a *Mycobacterium caprae* isolate (SRR761662) as the outgroup. (**b**) Time-scaled maximum-likelihood phylogenetic tree of 316 Eu2 isolates.

The 74 isolates from this study were combined with 53 published isolates from Argentina, Brazil, Paraguay and Uruguay to reconstruct a phylogenetic tree (Fig. 5a) that was rooted with *M. caprae* as the outgroup. The three CCs contained in the dataset, Eu1, Eu2 and unknown4, segregated into distinct lineages. The first published Eu2 genome from Brazil, SP38, sits amongst the Eu2 isolates from this study. Eu1 isolates from this study are both ancestral to and derived from Eu1 isolates from Uruguay and Argentina, suggesting two-way transmission between Brazil and neighbouring countries for isolates from this CC in RS.

In order to estimate a date of introduction for CC Eu2 into RS, the 64 sequenced Eu2 isolates from this study were combined with 252 published Eu2 isolates to build a time-scaled phylogenetic tree for the entire CC (Fig. 5b). The estimated age of the MRCA for Eu2 was 1762 AD [95 % confidence interval (CI): 1725–1795]. All of the RS Eu2 isolates formed a clade with two recent French isolates; the estimated date of the last common ancestor between the French isolates and the Brazilian Eu2 isolates was 1797 AD [95 % CI: 1759–1829] with the date of the MRCA of the Brazilian isolates being 1864 AD [95 % CI: 1837–1885] (Fig. 5b). These dates imply a possible date of introduction of Eu2 into RS in the middle of the 19th century. The estimated substitution rate for the Eu2 dataset was 0.83 SNPs per genome per year.

## Discussion

To date, this is the largest WGS-based study of *M. bovis* in Brazil and South America and included 74 isolates sourced from infected cattle in RS, southern Brazil. WGS genotyping identified 11 distinct spoligotypes including four new profiles and two CCs, Eu1 and Eu2. Four genetic lineages were assigned using fastBAPS. The overlap of the spatial distributions of these four fastBAPS clusters and the overlap of the within-herd and between-herd pairwise SNP distances, as well as the lack of association identified in two of the fastBAPS clusters using Mantel tests, showed that there is little spatial clustering of the genetic lineages circulating in this region. This was further confirmed by the presence of multiple genetic lineages, shown by high levels of genetic diversity in the majority of herds in the study dataset. Using thresholds of five and 15 SNPs, both putative within- and between-herd transmission events could be identified, indicating relatively recent long-distance transmission that is probably due to cattle movements. The inclusion of previously published isolates from Argentina, Brazil, Paraguay and Uruguay showed some evidence for the exchange of Eu1 between RS and Uruguay or Argentina. Dating analysis using a global Eu2 dataset suggested that Eu2 was introduced into southern Brazil sometime in the middle of the 19th century.

SB0295 was the most commonly observed spoligotype in this study, and is considered the second most frequently observed in Brazil [82]. Recently, this spoligotype was reported in northern Brazil [30], and had already been described in the states of Paraíba [83], Mato Grosso do Sul, Santa Catarina [84, 85], São Paulo [86], Bahia [87], Mato Grosso and Goiás [88]. SB0121 was the second most predominant spoligotype, and was also prevalent in previous studies in states of different Brazilian regions, including RS in southern Brazil [89]; São Paulo and Minas Gerais in south-eastern Brazil [84, 86]; Mato Grosso do Sul, Mato Grosso and Goiás in the midwest [84, 88]; and Bahia and Paraíba in the north-east [84, 87].

In a previous study, Eu1 was shown to be distributed across the Americas, with a high frequency in Argentina, Chile, Ecuador, Mexico and North America. In this study, Eu1 was found in 13.5 % (10/74) of samples, which is similar to previous estimates of prevalence in Brazil that ranged between 14.4 % (13/90) and 16 % (6/36) [83, 84]. The prevalence of Eu2, found in 64/74 (86.5 %) of isolates in this study, was similar to the prevalence identified in the states of Minas Gerais, São Paulo, Mato Grosso, Mato Grosso do Sul, Goiás, Tocantins and Pará, where the prevalence was 81.1 % (73/90) [90].

Given the presence of two more distantly related CCs within the dataset, the high level of genetic diversity observed between isolates from different animals was not unexpected. This high level of diversity was also reflected in the pairwise SNP distances observed in isolates from the same herds, in particular herds C and D (Fig. 3c). The analysis of within-host diversity identified isolates from the same animal that were up to 26 SNPs apart, highlighting the likelihood of these animals suffering from mixed infections, higher rates of within-host evolution or long-term infections that are not being detected. This level of within-host diversity is similar to that observed in cattle in Ethiopia [91], although further analyses of multiple isolates from the same animals in other settings will be required to see if this is common to all animals infected with bTB. Examination of the pairwise SNP diversity amongst the defined fastBAPS clusters showed that there was much less diversity amongst cluster 1, which comprised Eu1 isolates, when compared to the other three clusters comprising Eu2 isolates which had similar levels of diversity (Fig. 3b). Given the limited sampling and the close geographical proximity of the farms from which the cluster 1 isolates were collected, it is difficult to draw any strong conclusions but these results suggest that the local population of Eu1 has had less time to evolve and diversify and has possibly been circulating for a shorter time than Eu2.

We observed no correlation between genetic and spatial distance for fastBAPS clusters 1 and 4 but there was a stronger, significant correlation in clusters 2 and 3. Cluster 2 is almost entirely formed by six Jersey cows from a herd in the municipality of Teutônia (herd A) and six Holstein cows from two herds in Arroio do Tigre, approximately 177 km away, and belonging to the same mesoregion (Centro Oriental Rio-Grandense). The six Jersey cows from herd A were moved from the city of Candelária, 57 km from Arroio do Meio, 8 months previously. This suggests that there was a possible long-distance transmission of a lineage or lineages persisting in herds surrounding Arroio do Tigre and Candelária. The strong correlation in cluster 3 is probably due to the close geographical proximity of herds C and D (12 km apart) comprising beef cattle, that also share the same owner (see below), and herds E and F (1.1 km apart) which are dairy herds and share a stream between them. Whilst there are no official records of cattle movements between the dairy herds E, F and L, there is a strong local culture of animal replacements in dairy herds from nearby farms which are often unreported.

As there is currently no consensus as to the most appropriate SNP threshold to apply to defining transmission clusters in either bTB or *M. tuberculosis sensu stricto* [92–94], we used two different thresholds. Given the very slow mutation rate of *M. bovis* (0.5–1 SNP per genome every 2 years) [95], a threshold of 15 SNPs was chosen as it would potentially identify older transmission events and provide an indication of how likely transmission, recent or otherwise, could be identified using WGS. Using this threshold, we identified a limited number of transmission events including both within- and between-herd transmission (Fig. 4a). Assuming recent transmission (within the last 5 years) is indicated by a pairwise SNP threshold of five SNPs or fewer, we could find evidence for recent within-herd transmission in three of the 13 herds (A, F, J) and evidence of between herd-transmission between herds C and D (Fig. 4b). Farms C and D belong to the same owner: farm C is for cow/calf operations, and provides weaned calves for fattening operations on farm D.

The spatial overlap of the fastBAPS clusters, high within-herd genetic diversity, and the general lack of an association between genetic and spatial distance gives strong evidence for the *M. bovis* population in this region being well mixed and probably maintained by short- and long-distance cattle movements. In the majority of countries where bTB is prevalent, there tends to be strong geographical localization of *M. bovis* genotypes such as VNTR and spoligotypes [95–97]. This localization is typically attributed to infection from contiguous herds, the presence of a wildlife reservoir such as the Eurasian badger in Great Britain and the wild boar in mainland Europe, and short-range movements of infected animals between herds [96, 98, 99]. This pattern is consistent across countries with very different *M. bovis* populations such as Great Britain, New Zealand and the USA [95–97]. One notable exception to this is Uruguay where clusters of infection change location from year to year, suggesting long-distance transmission [100]. Given the small sample size in our study, we can only speculate on the differences observed between RS and other settings. The state of RS is located in the extreme south of Brazil, and has environmental and cultural peculiarities that make it distinct from other states in Brazil; it has severe winters and the areas for livestock production are not vast, with consequent specificities in livestock production [101]. This means that there are localized areas of livestock production and dairy farming rather than a contiguous area across the state with the majority of infected herds concentrated in the north. We were able to identify processes associated with geographical localization, such as transmission occurring between contiguous herds, herds with the same owner and herds within the same region, but given the distribution of our fastBAPs clusters a higher level of long-distance transmission is probably helping to homogenize the *M. bovis* population across the state with little or no geographical clustering being observed. Additionally, whilst there are confirmed cases of bTB in wild boar in RS [102], to date no WGS has been done on these isolates to assess their similarity to local bovine isolates, so it would be premature to speculate on the role wild boar may play in maintaining bTB reservoirs in RS.

Our temporal analysis of the global Eu2 collection using BEAST estimated the substitution rate of Eu2 as 0.83 SNPs per genome per year, which is in line with previous estimates of *M. bovis* substitution rates [37, 95, 103]. Two previous studies have estimated the Eu2 MRCA date as part of wider *M. bovis* lineage dating analyses using different software and evolutionary models. Using a relaxed clock and constant population size model in BEAST, Zimpel *et al*. [27] estimated the MRCA of 183 Eu2 isolates and five closely related isolates to fall between 355 AD and 1673 AD, although the exact date of the divergence between the Eu2 isolates and the five related isolates was not provided. Loiseau *et al*. [104] used a combination of various models in BEAST as well as LSD to estimate the date of the Eu2 MRCA as being between 1416 and 1705, although the dataset they used only included 17 Eu2 isolates due to the computational restraints typical to using BEAST. Our analysis estimated the date for the Eu2 MRCA to be 1762 with narrow 95 % CIs of approximately 30 years either side of this date. Although this date falls outside the upper range of the previous two estimates, our larger dataset of 316 isolates, the evidence of strong temporal signal (Figs S2 and S3) and comparatively narrow CIs mean that we can be confident about the date we obtained.

Broadly speaking, the lineages observed in the Eu2 phylogeny are specific to a single country or neighbouring countries such as Mexico and the USA that have extensive cattle trade. With the caveat of limited sampling from countries such as Canada, this suggests that inter-country trade is not a major source of infection, particularly in North America. For example, the clade composed of SB0265 isolates, with the exception of the outgroup from Canada, is exclusively composed of isolates from the USA. The estimated date of the MRCA of the entire clade is 1954 [95 % CI: 1944–1963] and of the American isolates is 1968 [95 % CI: 1961–1974; Fig. 5b]. This lineage is associated with outbreaks in farmed cervids and cattle in Canada and states such as Nebraska, Indiana and Michigan [25].

The state of RS was originally located in Spanish territory, to the west of the area covered by the Treaty of Tordesillas. The first Europeans to colonize the state were Jesuit priests, who founded missions and imported cattle from the Iberian Peninsula where Eu2 is the predominant genotype [105]. After the expulsion of the Jesuits in 1759, the cattle became wild and spread throughout the south of Brazil, Uruguay and Argentina. These cattle, known as the creole breed, formed the basis of the beef industry in RS in the 18th century. To improve this industry, the government began to import British breeds in the middle of the 19th century, directly from the UK, or via Argentina and Uruguay [70]. The estimated date of introduction of Eu2 into southern Brazil sometime in the middle of the 19th century is consistent with this re-stocking rather than it already being present in the older cattle populations. The MRCA date for the entire CC, 1762, would also support this. Whilst we did not perform any temporal analysis on the Brazilian Eu1 isolates, it is possible this CC, found at a high frequency in former colonies and trading partners of the UK, including Argentina and Uruguay, was also introduced into southern Brazil at this time [106]. However, the much lower diversity observed in the Eu1 isolates compared to those from Eu2 suggest a much more recent date of introduction (Fig. 3b). Further sampling and sequencing alongside a robust temporal analysis of the global Eu1 population would be required to confirm this.

A limitation of this study was the small sample size compared to the number of infected cattle in the region, estimated to be in the region of 84 000 head in 2013. Although this was due to limited available resources, clearly this hampered the detection of transmission events. However, we were still able to characterize significant diversity within the herds sampled and provide the first WGS-based description of the population structure of *M. bovis* in southern Brazil. Even with the limited sampling, we were able to detect within- and between-herd transmission at different SNP thresholds and found supporting evidence for one of these transmission events in the form of known cattle movements between farms with the same owner (herds C and D). Future studies could focus on collecting a larger number isolates from more herds with a focus on adjacent farms where the cattle movement data are available and bTB prevalence is consistently high.

Recently, the Brazilian bTB control programme (PNCEBT) [5] strategy has been revised: sanitation of outbreaks will be mandatory based on the classification of the federative units in relation to the degree of risk for bTB, and this was established in Normative Instruction 10/17 [5, 107]. With this new regulation, bTB state programmes can define strategies for eradicating the disease, based on a system for detecting bTB lesions in slaughterhouses and tracing back to the farm of origin, for sanitation of outbreaks. Evidently, surveillance for eradication must be supported by tools that allow an accurate assessment of the diversity and local persistence of outbreaks of bTB, as well as their dissemination, and WGS can be a useful and more reliable tool than traditional genotyping methods, as spoligotyping and MIRU-VNTR tend to be influenced by homoplasy [39, 108]. The results of this study show that genome sequencing has the potential to provide novel, detailed insights into local bTB transmission dynamics and should be considered as a useful tool for helping to control bTB transmission in RS.

## Supplementary Data

Supplementary material 1

Supplementary material 2
