# Supplementary material for: Genomic and temporal analyses of Mycobacterium bovis in southern Brazil
Source: Microb Genom. 2021 May 20;7(5):000569. doi: 10.1099/mgen.0.000569 (PMC8209730; doi:10.1099/mgen.0.000569)
Supplement: Supplementary material 2 [file mgen-7-0569-s002.pdf]

***Mycobacterium bovis* DNA extraction for genomic sequencing.**

DNA extractions from colonies suggestive of M. bovis for genomic sequencing were performed according to the protocol of van Embden et al. [45], with modifications. Initially, for inactivation, 2-3 colonies were resuspended in 400 µl TE buffer (10 mM Tris-HCI and 1 mM ethylenediaminetetraacetic acid - EDTA, pH 8.0) and heated in a water bath 80°C for 30 minutes. Subsequently, 50 µl of lysozyme (10 mg/ml) was added and incubated at 37°C for one hour. Then, 75 µl 10% SDS (sodium dodecyl sulfate) and 10 µl proteinase K (10 mg/ml) were added, and incubated at 65°C for 10 minutes. Next, 100 µl of 5M NaCl (sodium chloride) and 100 µl of CTAB (cetyltrimethylammonium bromide) were added, followed by stirring and incubation at 65°C for 10 minutes. After that, 750 µl chloroform/isoamyl alcohol (24: 1) was added, stirred and centrifuged at 12000 rpm for 5 minutes. The aqueous phase (surface) was transferred to another tube, 450 µl isopropanol was added and incubated at -20 ° C for 30 minutes and then centrifuged again at 12000 rpm for 15 minutes at room temperature. After that, the supernatant was discarded, 1 mL of ice-cold ethanol (70%) was added and, after centrifugation at 12000 rpm for 5 minutes, the supernatant was discarded again. After drying the tube by evaporation at room temperature, the DNA was resuspended in 20 µl TE buffer and stored in a freezer at –20°C. The quality and concentration of the extracted DNAs were evaluated by a Qubit fluorimeter (Invitrogen).

Supplementary Table 1: Model performance based on marginal likelihood estimates (MLE) and Bayes factors.

Model	log MLE	log Bayes Factor	Strength of Evidence (Kass & Raftery, 1995)
**Relaxed constant**	-5902114.13664	-	-
**Relaxed exponential**	-5902120.45420	6.31756478734	Very strong
**Strict exponential**	-5902186.47077	72.3341384875	Very strong
**Strict constant**	-5902188.01921	73.8825739417	Very strong

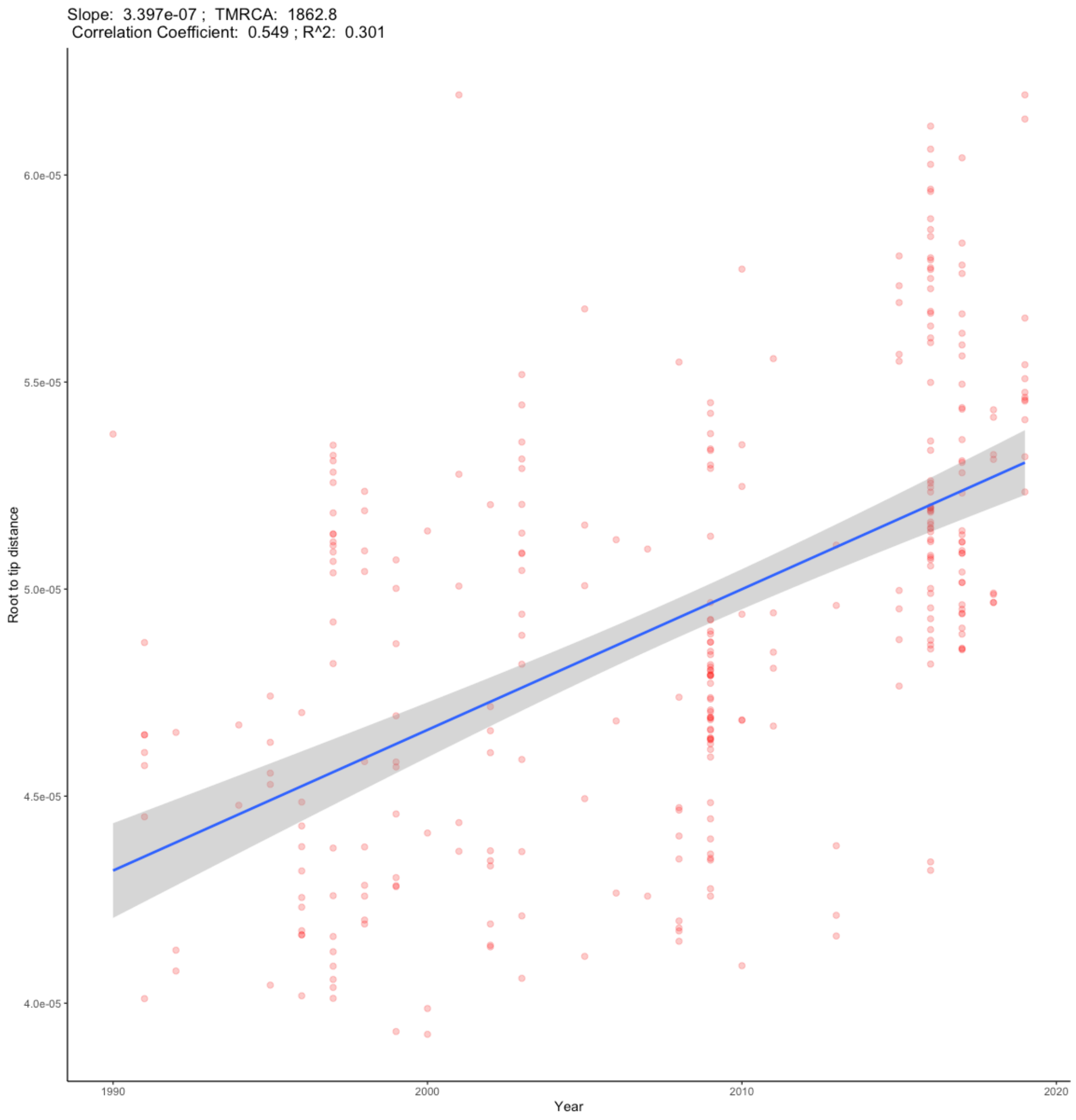

Supplementary Figure 1: Root to tip distances plotted against sampling dates for Eu2 isolates.

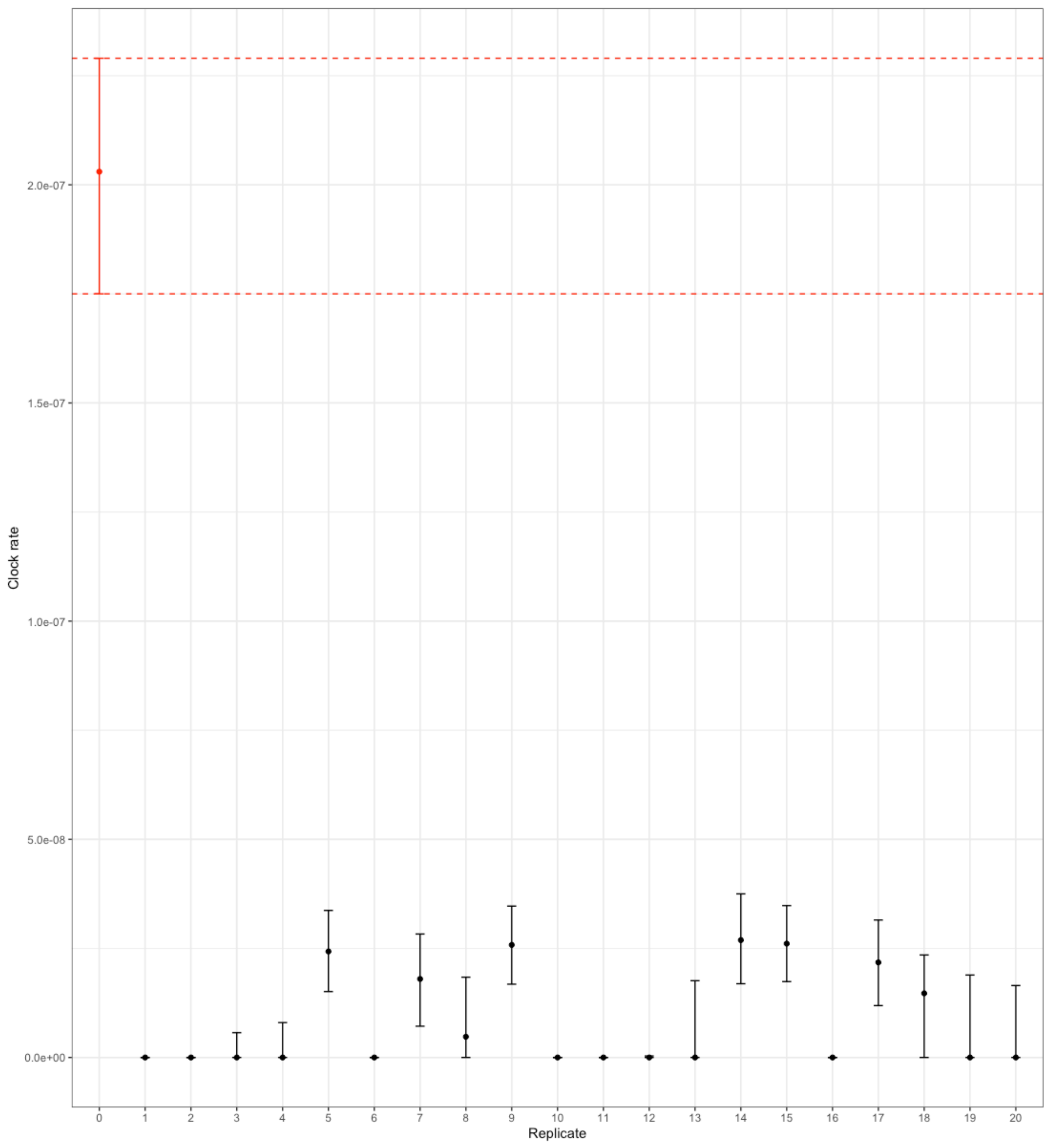

Supplementary Figure 2: Date randomization (DTR) analysis in BEAST. Estimated substitution rates (mean and highest posterior density) shown in red for the observed dataset and black for the randomized datasets.
